# Retrospective analysis of predictors for significant hepatic inflammation in metabolic-associated fatty liver disease with chronic hepatitis B

**DOI:** 10.7717/peerj.21186

**Published:** 2026-05-06

**Authors:** Long Hao Xu, Run Yan He, Yan Neng Kang, Heng Hong Lin, Jia En Yang, Hua Xiao Chen, Xue Ting Huang, Xiao Lu Wu, Bao Hui Pei, Chao Zhou, Min Wang

**Affiliations:** 1 Department of Hepatology, Xiamen TCM Hospital Affiliated to Fujian University of Traditional Chinese Medicine, Xiamen, Fujian Province, China; 2 Department of Hepatology, Xiamen Humanity Hospital, Xiamen, Fujian Province, China; 3 Heshan District Health Service Center, Xiamen, Fujian Province, China; 4 Department of Liver Disease, 5th Medical Center of Chinese PLA General Hospital, Beijing, China

**Keywords:** Metabolic-associated (non-alcoholic) fatty liver disease, Hepatitis B virus infection, Significant hepatic inflammation

## Abstract

**Background:**

The disease burden of metabolic dysfunction–associated fatty liver disease (MAFLD, non-alcoholic) combined with hepatitis B virus (HBV) infection is rising. Hepatic inflammation is a common pathway in liver disease progression, and evaluating its risk factors has clinical significance. In the context of concomitant MAFLD and HBV infection, hepatic inflammation may represent a key pathological pathway driving disease progression, making the assessment of liver inflammation crucial for controlling disease advancement.

**Objective:**

To identify independent risk factors for significant hepatic inflammation (grade ≥ 2) in patients with MAFLD (non-alcoholic) combined with HBV infection.

**Methods:**

This retrospective study included 447 treatment-naïve patients with metabolic dysfunction–associated fatty liver disease (non-alcoholic) and chronic hepatitis B who were diagnosed at Xiamen Hospital of Traditional Chinese Medicine between 2018 and 2024. Patients were stratified by body mass index (BMI) as normal weight (BMI < 25 kg/m^2^; *n* = 168), overweight (25 ≤ BMI ≤ 30 kg/m^2^; *n* = 234), and obese (BMI > 30 kg/m^2^; *n* = 45) to compare inflammatory differences across BMI categories. Liver biopsy was used as the histological reference standard, and multivariable logistic regression was performed to further identify the independent risk factors for significant hepatic inflammation (grade ≥ 2).

**Results:**

Baseline characteristics revealed statistically significant differences among BMI groups in controlled attenuation parameter (CAP), liver stiffness measurement (LSM), alanine aminotransferase (ALT), aspartate aminotransferase (AST), gamma-glutamyl transferase (GGT), uric acid (UA), glucose (GLU), hepatitis B surface antigen (HBsAg), hepatitis B e-antigen (HBeAg) positivity rate, and non-invasive indices APRI, GPR, and AAGP (all *P* < 0.05). A correlation was observed between BMI groups and hepatic inflammation grading (*P* < 0.05), persisting even after dichotomizing inflammation grades into G1 versus G2–4 (*P* < 0.05). Multivariate logistic regression analysis demonstrated that BMI (OR = 1.12, 95% CI [1.01–1.26]), LSM (OR = 1.34, 95% CI [1.17–1.53]), ALT (OR = 1.01, 95% CI [1.01–1.02]), and HBV DNA (OR = 1.32, 95% CI [1.16–1.51]) were independent risk factors for significant hepatic inflammation (G ≥ 2) (all *P* < 0.05).

**Conclusions:**

This clinical study was conducted based on histopathological findings and demonstrated that BMI, LSM, and HBV DNA are independent risk factors for the progression of hepatic inflammation in patients with MAFLD and HBV infection, whereas ALT served as an independent non-invasive predictor of this outcome. These findings provide a novel non-invasive approach for clinicians to assess the degree of hepatic inflammation when liver biopsy results are unavailable.

## Introduction

Metabolic dysfunction-associated fatty liver disease (MAFLD) is a chronic metabolic stress-related liver disease caused by nutrient excess and insulin resistance, characterized predominantly by hepatocellular steatosis, and places greater emphasis than non-alcoholic fatty liver disease (NAFLD) on its close association with metabolic dysfunction (Fan et al., 2024). The systemic metabolic dysfunction associated with MAFLD not only accelerates liver disease progression but also markedly increases the risk of cardiovascular disease, renal disease, and malignancies through mechanisms such as lipotoxicity and inflammation (Fan et al., 2024; Quek et al., 2023). Therefore, MAFLD represents not only a hepatic manifestation of metabolic dysfunction but also an independent risk factor for cardiovascular health (Alebna et al., 2024; Mantovani et al., 2023).

Hepatitis B virus (HBV) infection remains a major global public health concern (Alberts et al., 2022). With changes in lifestyle and the increasing prevalence of metabolic syndrome, the prevalence of concomitant MAFLD and HBV infection has risen markedly (Xu, Nguyen & Li, 2024). Although viral hepatitis is a common cause of liver-related mortality, MAFLD represents the fastest-growing contributor to liver-related complications (Golabi et al., 2021). The presence of MAFLD is an independent risk factor for progression to cirrhosis and hepatocellular carcinoma in patients with chronic hepatitis B (CHB) (Zhang et al., 2025).

Hepatic inflammation, triggered by persistent liver injury and hepatocyte death, is regarded as a common pathway in the progression of various chronic liver diseases and plays a pivotal role in hepatocellular malignant transformation and the development of hepatocellular carcinoma (HCC) (Chen et al., 2021; Jiang et al., 2021; Sobolewski et al., 2020). MAFLD promotes the release of proinflammatory cytokines and initiates inflammatory cascades, while chronic HBV infection sustains hepatic inflammation through immune-mediated responses; the convergence of metabolic inflammatory burden and virus-related immune inflammation may remodel the hepatic immune microenvironment, thereby exacerbating hepatocellular injury, accelerating fibrosis progression, and influencing the risk of HCC development (Huang & Liu, 2023; Liu, Seto & Yu, 2024; Zhu et al., 2025). Therefore, assessment of hepatic histological inflammation in patients with concomitant MAFLD and HBV infection is of substantial clinical importance.

Although liver biopsy remains the gold standard for assessing hepatic inflammation, its invasive nature and potential risk of complications limit its widespread use in clinical practice. Therefore, exploring non-invasive approaches based on routinely available clinical parameters to assess the risk of hepatic inflammation has practical value for improving risk stratification and clinical management. In this study, 447 patients with concomitant MAFLD and HBV infection were enrolled, histopathological findings were used as the reference standard, differences in hepatic inflammation among MAFLD subtypes defined by BMI were compared, and independent risk factors for inflammation progression were identified using regression analyses to inform individualized treatment strategies.

## Materials and Methods

### Patients

**Figure 1:**
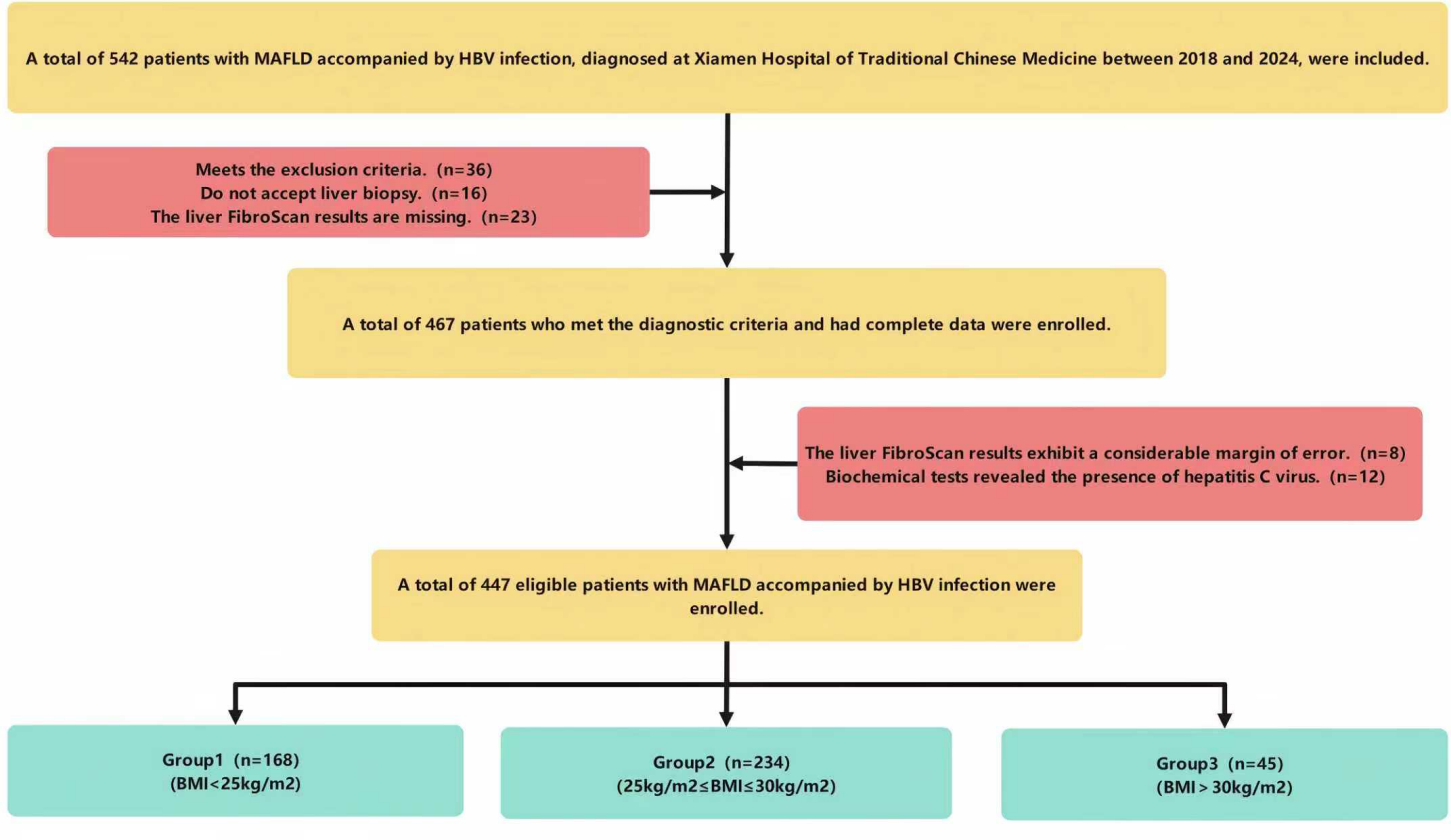
Flow diagram of patient selection process.

This study included patients with MAFLD (without alcohol consumption) and HBV infection at Xiamen Hospital of Traditional Chinese Medicine between 2018 and 2024. None had received prior antiviral therapy for HBV. The study protocol was approved by the Ethics Committee of Xiamen Hospital of Traditional Chinese Medicine (Approval No. 2025-X075-01) with a waiver of informed consent. The patient screening process is presented in Fig. 1. A total of 542 patients were initially identified. Of these, 36 patients who met the exclusion criteria were excluded. Among the remaining candidates, 16 declined liver biopsy and 23 were excluded due to missing FibroScan data. After these exclusions, 467 patients remained eligible for further assessment. Among these 467 patients, eight were excluded because of unreliable FibroScan results, and 12 were excluded after subsequent biochemical testing revealed hepatitis C virus co-infection. Ultimately, 447 patients were included in the final analysis. Participants were stratified into three groups according to the World Health Organization (WHO) BMI classification: normal weight (BMI < 25 kg/m^2^, *n* = 168), overweight (25 ≤ BMI ≤ 30 kg/m^2^, *n* = 234), and obese (BMI > 30 kg/m^2^, *n* = 45).

### Diagnostic criteria for MAFLD and CHB

Diagnostic criteria: Patients met the diagnostic standards outlined in both the Guidelines for the Prevention and Treatment of CHB (You et al., 2023) and the Guidelines for the Prevention and Treatment of MAFLD (without alcohol consumption) (Fan et al., 2024).

### Inclusion criteria

(1) No gender restriction; aged 18–70 years. (2) Patients had never received antiviral therapy for HBV, had undergone liver biopsy, and had available histological staging results for liver fibrosis. (3) Completion of all required laboratory tests and examinations within one week prior to liver biopsy.

### Exclusion criteria

Presence of any of the following: (1) Coexisting viral hepatitis (non-HBV) or other chronic liver diseases. (2) Daily alcohol intake exceeding 20 g (male) or 10 g (female). (3) Comorbid pulmonary, renal, gastrointestinal, circulatory, or psychiatric disorders. (4) Pregnancy or lactation, or individuals with allergic predisposition. (5) Use of medications known to affect lipid metabolism or liver function within the past 6 months. (6) Prior anti-HBV antiviral therapy.

### Liver biopsy

Under ultrasound guidance, liver biopsy was performed using a 16G Bard needle. Adequate specimens were defined as tissue lengths of ≥ 15 mm containing at least six portal tracts to ensure reliable pathological assessment. Samples were fixed in 10% neutral-buffered formalin, embedded in paraffin, and stained with hematoxylin and eosin as well as reticulin. Histologic slides were independently reviewed by two pathologists at our hospital, and any discrepancies were adjudicated by a third senior pathologist. Hepatic inflammatory activity was graded according to the Scheuer scoring system, which categorizes inflammation into four grades (G1–G4).

This study enrolled patients with MAFLD (non-alcoholic) and concomitant HBV infection who were treatment-naïve and had never received antiviral therapy for HBV before enrollment. Because of the complex mechanisms underlying liver injury in this population, non-invasive approaches such as laboratory testing and imaging, although clinically valuable, remain limited in accurately distinguishing inflammation from fibrosis and in determining histological stage. Current guidelines indicate that liver biopsy may be performed in the presence of multiple liver-related etiologies (Cornberg et al., 2025), which supports its necessity in this study population. As the established gold standard for diagnosis and staging, liver biopsy provides direct and reliable pathological evidence of hepatic inflammation under the coexistence of multiple etiologies, thereby offering an objective basis for diagnostic confirmation and prognostic assessment.

### Fibroscan

Fibroscan was used to assess liver stiffness measurement (LSM). The procedure was performed by a certified and formally trained technician, strictly following the operating manual. To ensure measurement accuracy, all patients were prepared according to the following protocol: patients were required to fast for at least 3 h before the measurement to reduce the potential impact of food intake on liver stiffness values. During the examination, patients were positioned supine with their right arm extended and abducted to facilitate probe placement. The measurement site was chosen between the right anterior and midaxillary lines, in the 7th to 9th intercostal spaces, which effectively minimizes inaccuracies due to rib interference. The probe was held perpendicular to the skin to ensure that the shear wave transmission was not affected by angle deviations, and the optimal liver region free of cysts, nodules, or hemangiomas was selected to avoid measurement errors caused by these structural variations. At the designated site, 10 consecutive measurements were performed for each patient, and all valid readings were recorded. Throughout the procedure, patients were instructed to maintain steady respiration to minimize the interference of respiratory movements on the results. A measurement was considered valid if, among the 10 readings, the standard deviation was less than one third of the median value and at least 60% of the readings were valid. Finally, the median of all measurements was calculated to represent the patient’s liver stiffness value.

### Non-invasive tests

Non-invasive indices were computed as follows.

APRI (AST to Platelet Ratio Index) was derived as the ratio of AST (IU/L) to the upper limit of normal AST (IU/L), divided by the platelet count (10^9^/L), and multiplied by 100 (Wai et al., 2003).

FIB-4 (Fibrosis-4 Index) was derived as age (years) multiplied by AST (U/L), divided by the product of platelet count (10^9^/L) and the square root of ALT (U/L) (Vallet-Pichard et al., 2007).

GPR (GGT-to-platelet ratio) was derived as the ratio of GGT (U/L) to the upper limit of normal (ULN) for GGT, divided by the platelet count (×10^9^/L), and multiplied by 100 (Wang et al., 2018).

The AAGP score was constructed from four variables—age, alanine aminotransferase (ALT), gamma-glutamyl transferase (GGT), and platelet count (PLT)—with points assigned as follows: age (years), ≤ 30 = 0, >30 and ≤ 40 = 2, >40 = 3; ALT (U/L), ≤ 20 = 0, >20 and ≤ 30 = 1, >30 = 4; GGT (U/L), ≤ 50 = 0, >50 = 2; platelet count (× 10^9^/L), >200 = 0, >100 and ≤ 200 = 1, ≤ 100 = 3. The total AAGP score is the sum of these components, ranging from 0 to 12 (Li et al., 2018).

### Statistical analysis

Statistical analysis was performed using SPSS 25.0 software (IBM Corp., Armonk, NY, USA). Categorical data are expressed as frequency count and percentage, while non-normal data are presented as median (Q1, Q3). Intergroup differences were analyzed using the Kruskal-Wallis H test, Spearman’s rank correlation, and Kendall’s tau-b correlation. Univariate and multivariate logistic regression analyses were applied to identify independent risk factors for significant liver inflammation (G ≥ 2) in MAFLD (non-alcoholic) patients with HBV infection. A *P* value <0.05 indicated statistical significance.

## Results

### Baseline characteristics of 447 patients with MAFLD (non-alcoholic) and HBV coinfection

This study included 447 participants stratified into three BMI-based groups: BMI < 25 kg/m^2^ (*n* = 168); 25 ≤ BMI ≤ 30 kg/m^2^ (*n* = 234); BMI > 30 kg/m^2^ (*n* = 45). Baseline characteristics revealed statistically significant intergroup differences (*P* < 0.05) in CAP (*H* = 38.34, *P* < 0.001); LSM (*H* = 13.28, *P* = 0.001); ALT (*H* = 14.51, *P* < 0.001); AST (*H* = 11.72, *P* = 0.003); GGT (*H* = 7.81, *P* = 0.020); UA (*H* = 10.71, *P* = 0.005); GLU (*H* = 10.88, *P* = 0.004); HBsAg (*H* = 6.23, *P* = 0.044); HBeAg (+) (*H* = 9.17, *P* = 0.010); APRI (*H* = 7.64, *P* = 0.022); GPR (*H* = 13.92, *P* < 0.001); and AAGP (*H* = 10.61, *P* = 0.005). No significant differences were found in age (*H* = 3.42, *P* = 0.181); PLT (*H* = 0.59, *P* = 0.746); GLB (*H* = 0.17, *P* = 0.919); ALP (*H* = 0.75, *P* = 0.688); TBIL (*H* = 0.26, *P* = 0.879); TG (*H* = 0.87, *P* = 0.647); CHO (*H* = 0.47, *P* = 0.791); LDL (*H* = 3.32, *P* = 0.190); HBV DNA (log10) (*H* = 5.44, *P* = 0.060); TyG index (Yin et al., 2024) (*H* = 2.51, *P* = 0.285); FIB-4 (*H* = 0.05, *P* = 0.977). The detailed baseline characteristics are summarized in Table 1.

**Table 1:** Baseline characteristics of 447 patients with MAFLD (non-alcoholic) and HBV coinfection.

**Variables**	**Total**	**BMI < 25 kg/m** ^**2**^	**25 ≤ BMI≤30 kgm** ^**2**^	**BMI > 30 kg/m** ^**2**^	**H**	***P* value**
Age (year)	40.00 (34.00, 46.00)	39.00 (32.75, 48.00)	41.00 (34.00, 46.00)	38.00 (33.00, 42.00)	3.42	0.181
BMI (kg/m2)	25.97 (24.47, 28.06)	24.22 (22.89, 24.56)	27.00 (26.00, 28.25)	32.19 (30.89, 33.91)	357.89	<0.001
CAP (dB/m)	272.00 (247.25, 303.00)	261.50 (236.00, 280.25)	278.00 (252.00, 310.00)	310.00 (276.00, 350.00)	38.34	<0.001
LSM (kPa)	6.90 (5.30, 9.50)	6.40 (5.07, 8.93)	7.10 (5.60, 9.30)	8.00 (6.30, 12.60)	13.28	0.001
PLT (10^9^ g/L)	218.00 (182.00, 258.00)	216.50 (184.00, 253.00)	218.50 (177.00, 258.75)	220.00 (184.00, 265.00)	0.59	0.746
PT (s)	12.70 (12.10, 13.20)	12.70 (12.17, 13.20)	12.60 (11.90, 13.20)	13.10 (12.60, 13.40)	9.59	0.008
GLB (g/L)	29.00 (27.00, 32.00)	29.00 (27.00, 32.00)	29.00 (27.00, 32.00)	30.00 (26.00, 32.00)	0.17	0.919
ALT (U/L)	54.00 (33.00, 99.50)	53.00 (30.67, 92.25)	52.00 (32.25, 92.75)	88.00 (58.00, 146.00)	14.51	<0.001
AST (U/L)	33.00 (23.85, 58.00)	32.00 (23.00, 51.25)	32.00 (23.35, 56.75)	45.70 (33.00, 98.00)	11.72	0.003
GGT (U/L)	39.00 (24.00, 66.40)	36.50 (23.00, 62.25)	39.00 (25.00, 66.50)	47.00 (33.00, 88.00)	7.81	0.020
ALP (U/L)	76.00 (64.00, 90.95)	75.00 (64.53, 88.00)	76.85 (64.00, 92.00)	78.00 (64.50, 101.00)	0.75	0.688
TBIL (μmol/L)	14.40 (11.20, 18.90)	14.50 (11.28, 18.83)	14.10 (10.95, 19.08)	15.00 (11.90, 18.40)	0.26	0.879
TG (mmol/L)	1.37 (1.04, 1.85)	1.30 (1.02, 1.77)	1.41 (1.06, 1.92)	1.38 (1.07, 1.66)	0.87	0.647
CHO (mmol/L)	4.97 (4.23, 5.70)	4.92 (4.21, 5.61)	5.00 (4.30, 5.70)	4.90 (4.20, 5.70)	0.47	0.791
LDL (mmol/L)	3.11 (2.55, 3.69)	3.03 (2.45, 3.64)	3.20 (2.59, 3.70)	3.15 (2.57, 3.74)	3.32	0.190
HDL (mmol/L)	1.12 (0.95, 1.34)	1.13 (0.94, 1.34)	1.14 (0.98, 1.38)	1.05 (0.92, 1.21)	5.89	0.050
UA (μmol/L)	396.00 (337.00, 457.50)	381.50 (333.00, 431.32)	397.75 (337.00, 466.58)	441.00 (371.00, 488.00)	10.71	0.005
GLU (mmol/L)	5.20 (4.80, 5.54)	5.08 (4.80, 5.45)	5.20 (4.80, 5.60)	5.38 (5.00, 5.80)	10.88	0.004
HBVDNA (log^10^ IU/mL)	4.00 (2.00, 6.00)	4.00 (2.00, 7.00)	3.00 (2.00, 5.25)	4.00 (2.00, 6.00)	5.44	0.060
HBsAg (ng/mL)	2,008.87 (322.13, 9,886.06)	3,084.36 (377.07, 23,129.98)	1,584.11 (322.13, 5,569.95)	2,274.92 (210.77, 10,205.96)	6.23	0.044
HBeAg(+) (n (%))	184 (41.16%)	56 (33.33%)	112 (47.86%)	16 (35.56%)	9.17	0.010
TyG index	1.27 (0.98, 1.62)	1.23 (0.97, 1.58)	1.29 (0.99, 1.66)	1.28 (1.02, 1.55)	2.51	0.285
APRI	0.39 (0.27, 0.76)	0.36 (0.26, 0.72)	0.38 (0.28, 0.71)	0.59 (0.33, 1.05)	7.64	0.022
FIB-4	0.89 (0.64, 1.30)	0.89 (0.62, 1.25)	0.89 (0.64, 1.31)	0.91 (0.65, 1.47)	0.05	0.977
GPR	0.34 (0.18, 0.55)	0.29 (0.17, 0.49)	0.32 (0.19, 0.54)	0.47 (0.35, 0.56)	13.92	<0.001
AAGP	7.00 (5.50, 8.00)	7.00 (4.00, 8.00)	7.00 (6.00, 8.00)	8.00 (7.00, 8.00)	10.61	0.005

**Notes.**

BMI body mass index CAP controlled attenuation parameter LSM liver stiffness measurement PLT platelet count PT prothrombin time GLB globulin ALT alanine aminotransferase AST aspartate aminotransferase GGT gamma-glutamyl transferase ALP alkaline phosphatase TBIL total bilirubin TG triglycerides CHO cholesterol LDL low-density lipoprotein HDL high-density lipoprotein UA uric acid GLU glucose HBV DNA hepatitis B virus DNA HBsAg hepatitis B surface antigen HBeAg hepatitis B e antigen TyG index triglyceride glucose index APRI aspartate aminotransferase to platelet ratio index FIB-4 fibrosis-4 score GPR gamma-glutamyl transpeptidase to platelet ratio AAGP AAGP algorithm

### Grading distribution of hepatic inflammation in patients with MAFLD (non-alcoholic) and HBV coinfection

The distribution of hepatic inflammation grades is presented in Table 2: G1 (*n* = 86; 19.2%), G2 (*n* = 290; 64.9%), G3 (*n* = 64; 14.3%), and G4 (*n* = 7; 1.5%). A significant correlation existed between BMI groups and inflammation grades (Spearman’s *ρ* = 0.108, *P* = 0.022), with preserved ordinal association (Kendall’s tau-b = 0.100, *P* = 0.022). When regrouping as G1 *versus* G2–G4 (*n* = 361, 80.8%), the BMI-grade correlation persisted (Spearman’s *ρ* = 0.095, *P* = 0.035) with maintained ordinal relationship (Kendall’s tau-b = 0.091, *P* = 0.035).

**Table 2:** Grading distribution of hepatic inflammation in patients with MAFLD (non-alcoholic) and HBV coinfection.

	**Total (*n* = 447)**	**Group1 (*n* = 168)**	**Group2 (*n* = 234)**	**Group3 (*n* = 45)**	***P value***
G1	86 (19.2%)	38 (22.6%)	45 (19.2%)	3 (6.7%)	0.022
G2	290 (64.9%)	107 (63.7%)	152 (65.0%)	31 (68.9%)	
G3	64 (14.3%)	21 (12.5%)	36 (15.4%)	7 (15.6%)	
G4	7 (1.5%)	2 (1.2%)	1 (0.4%)	4 (8.9%)	
≥G2	361 (80.8%)	130 (77.3%)	189 (80.7%)	42 (93.3%)	0.035

**Notes.**

G1 grading of hepatic inflammation at stage G1 G2 grading of hepatic inflammation at stage G2 G3 grading of hepatic inflammation at stage G3 G4 grading of hepatic inflammation at stage G4 CHB chronic hepatitis B MAFLD metabolic associated fatty liver disease

**Figure 2:**
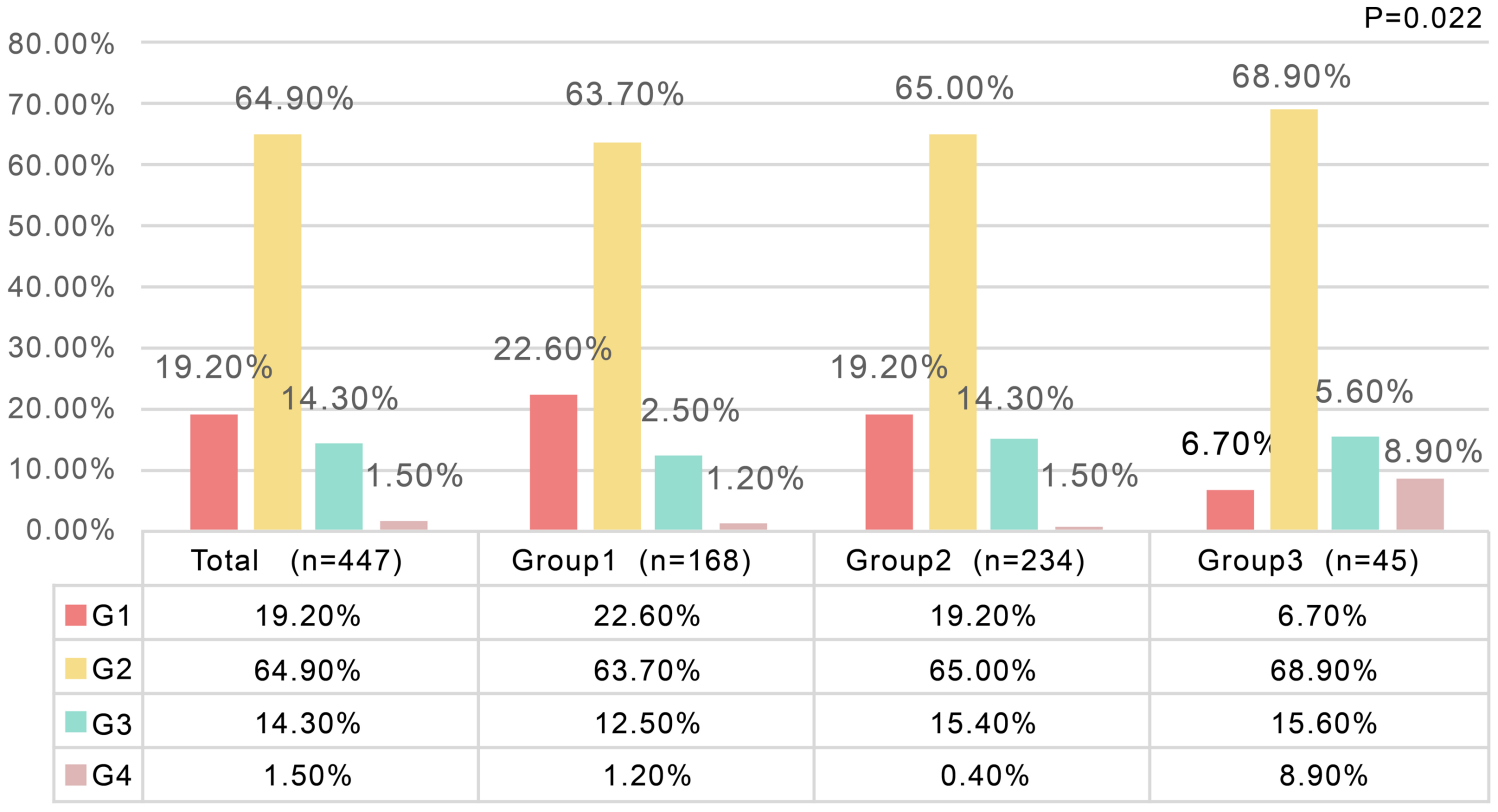
Grading distribution of hepatic inflammation in patients with MAFLD (non-alcoholic) and HBV coinfection. The bar plot presents the distribution of hepatic inflammation grades (G1–G4) for the overall cohort (*n* = 447) and stratified by BMI categories: Group 1 (BMI < 25 kg/m^2^, *n* = 168), Group 2 (25 ≤ BMI ≤ 30 kg/m^2^, *n* = 234), and Group 3 (BMI > 30 kg/m^2^, *n* = 45). Grade 2 was the predominant category across all groups (63.7–68.9%), while Grade 1 accounted for 19.2% overall, with a higher proportion observed in Group 1 (22.6%) compared with Group 3 (6.7%). Grade 3 comprised 14.3% of the total population and showed a slight increasing trend with higher BMI (12.5% in Group 1, 15.4% in Group 2, and 15.6% in Group 3). Grade 4 inflammation was relatively uncommon overall (1.5%) but was more frequently observed in Group 3 (8.9%) compared with Group 1 (1.2%) and Group 2 (0.4%). A statistically significant difference in inflammation grade distribution was observed among BMI groups (*P* = 0.022).

**Figure 3:**
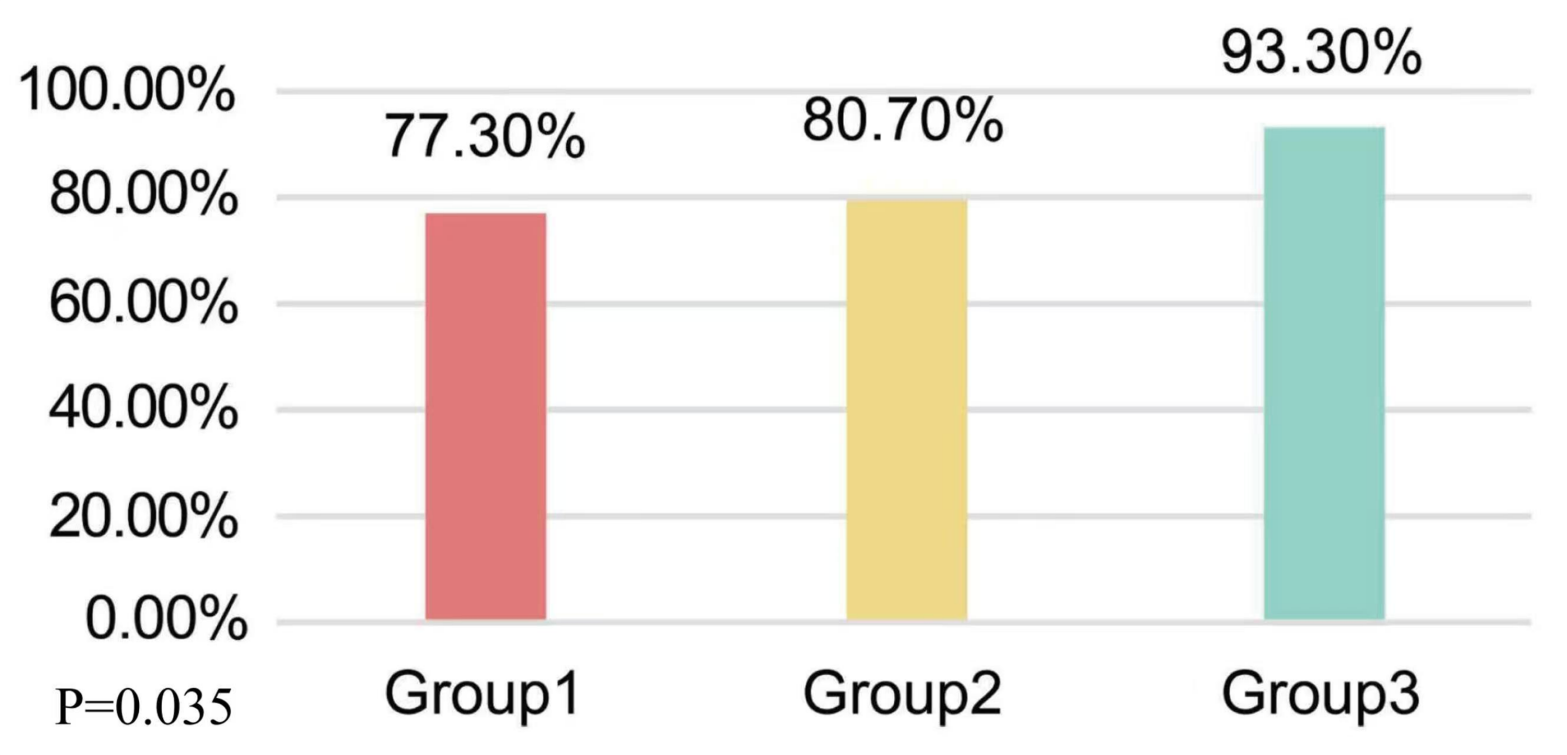
Distribution of MAFLD (non-alcoholic) patients with HBV infection and significant liver inflammation (Grade ≥ 2). The bar plot illustrates the prevalence of significant inflammation in each BMI group: Group 1 (BMI < 25 kg/m^2^, *n* = 168), 77.3%; Group 2 (25 ≤ BMI ≤ 30 kg/m^2^, *n* = 234), 80.7%; and Group 3 (BMI ¿30 kg/m^2^, *n* = 45), 93.3%. The increasing trend across the three BMI categories indicates a positive correlation between BMI and the presence of significant hepatic inflammation.

**Figure 4:**
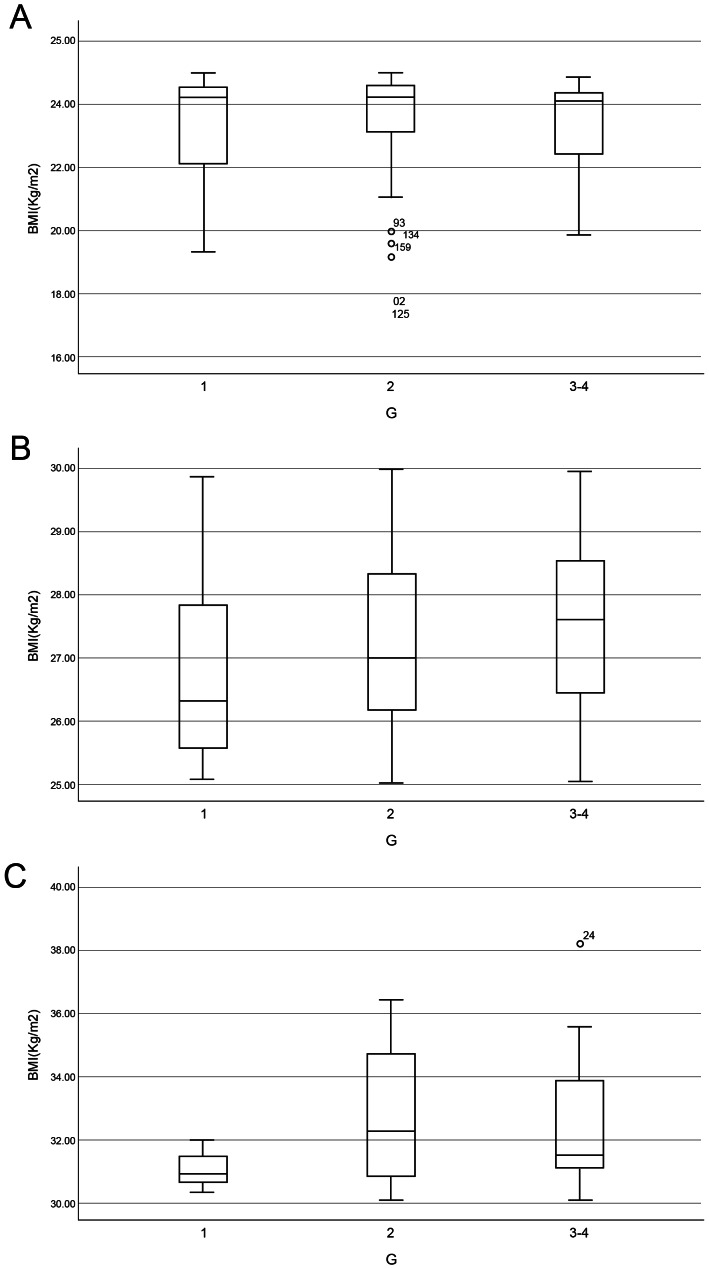
Box plot graphs illustrating the distribution of BMI among patients with different levels of liver inflammation within different groups. Box plots show BMI values stratified by inflammation grades (G1, G2, G3–4) within three BMI categories: (A) Group 1 (BMI < 25 kg/m^2^, *n* = 168), (B) Group 2 (25 ≤ BMI ≤ 30 kg/m^2^, *n* = 234), and (C) Group 3 (BMI > 30 kg/m^2^, *n* = 45). Each box represents the interquartile range (25th–75th percentile), the horizontal line indicates the median, whiskers extend to 1.5 × the interquartile range, and circles denote outliers. The plots illustrate that higher BMI groups tend to present with greater proportions of advanced hepatic inflammation.

The distribution of hepatic inflammation grades is further illustrated in Figs. 2–4. Figure 2 shows the overall pattern of inflammation grades. Figure 3 shows the proportion of patients with significant inflammation (Grade ≥ 2). Figure 4 shows the distribution of BMI across different inflammation grades within each BMI category.

### Independent risk factors for significant liver inflammation (G ≥ 2) in patients with MAFLD (non-alcoholic) and HBV coinfection

Univariate analysis showed that significant hepatic inflammation (G ≥ 2) in patients with MAFLD and HBV infection was associated with BMI, LSM, PLT, ALT, AST, GGT, high-density lipoprotein (HDL), HBV DNA, HBeAg positivity, and AAGP. After inclusion of these variables in multivariate regression, BMI (OR = 1.12, 95% CI [1.01–1.26], *P* = 0.040), LSM (OR = 1.34, 95% CI [1.17–1.53], *P* < 0.001), ALT (OR = 1.01, 95% CI [1.01–1.02], *P* < 0.001), and HBV DNA (OR = 1.32, 95% CI [1.16–1.51], *P* < 0.01) were identified as independent risk factors for significant hepatic inflammation (G ≥ 2) in patients with MAFLD and HBV infection. The results of the logistic regression analyses are summarized in Table 3.

**Table 3:** Independent risk factors for significant liver inflammation (G ≥ 2) in patients with MAFLD (non-alcoholic) and HBV coinfection.

	Univariable *OR* (95% *CI*)	*P* value	Multivariable *OR* (95% *CI*)	*P* value
BMI				
<30 kg/m^2^	1		1	
≥30 kg/m^2^	1.15 (1.06∼1.26)	0.001	1.12 (1.01∼1.26)	0.040
LSM				
<7.3 kPa	1		1	
≥7.3 kPa	1.40 (1.24∼1.59)	<0.001	1.34 (1.17∼1.53)	<0.001
PLT				
<350*10^9^ g/L	1			
≥350*10^9^ g/L	0.99 (0.99∼0.99)	0.006		
ALT				
<40 U/L	1		1	
≥40 U/L	1.03 (1.02∼1.04)	<0.001	1.01 (1.01∼1.02)	0.005
AST				
<40 U/L	1			
≥40 U/L	1.03 (1.02∼1.05)	<0.001		
GGT				
<60 U/L	1			
≥60 U/L	1.01 (1.01∼1.02)	0.001		
HDL				
<1.04 mmol/L	1			
≥1.04 mmol/L	0.38 (0.18∼0.80)	0.011		
HBVDNA				
<20 IU/mL	1		1	
≥20 IU/mL	1.34 (1.19∼1.50)	<0.001	1.32 (1.16∼1.51)	<0.001
AAGP				
<5	1			
≥5	1.42 (1.26∼1.60)	<0.001		
HBeAg(+)				
<0.5 PEIU/mL	1			
≥0.5 PEIU/mL	2.09 (1.30∼3.36)	0.002		
<40 U/L	1			
Patients with MAFLD (non-alcoholic) and HBV coinfection				
Group 1	1			
Group 2	1.23 (0.76∼2.00)	0.408		
Group 3	4.09 (1.20∼13.94)	0.024		

**Notes.**

BMI body mass index CAP controlled attenuation parameter LSM liver stiffness measurement PLT platelet ALT alanine aminotransferase AST aspartate aminotransferase GGT gamma-glutamyl transferase HDL high-density lipoprotein HBeAg hepatitis B e antigen AAGP AAGP algorithm HBV DNA hepatitis B virus DNA

## Discussion

Persistent hepatic inflammation, as a common pathological basis of multiple chronic liver diseases, can markedly accelerate the progression of liver fibrosis and end-stage liver disease (Friedman & Pinzani, 2022; Taru et al., 2024). The hepatic inflammatory status in patients with concomitant MAFLD and HBV infection is more complex than that observed in isolated MAFLD or CHB, and its determinants and clinical assessment require further elucidation. Currently, studies investigating factors associated with hepatic inflammation in patients with concomitant MAFLD and HBV infection remain limited (Chen et al., 2022). Our findings indicate that LSM, BMI, and HBV DNA are independent risk factors for significant hepatic inflammation (G ≥ 2) in patients with MAFLD and HBV infection, whereas ALT, as a non-invasive surrogate marker reflecting hepatocellular injury and inflammatory activity, demonstrates independent predictive value.

The final cohort comprised 447 patients, aged 19–70 years with a median age of 40 years. Of these, 80.8% (361/447) were male and 19.2% (85/447) were female. A total of 6.0% (27/447) had elevated blood pressure based on a single measurement (SBP/DBP ≥ 140/90 mmHg), 10.3% (46/447) had fasting glucose ≥ 6.1 mmol/L, 31.1% (139/447) had triglyceride levels ≥ 1.70 mmol/L, and 1.7% (8/447) met the diagnostic criteria for metabolic syndrome. In addition, 66.2% (296/447) were HBeAg-negative, and 38.3% (171/447) had normal liver function. MAFLD-associated comorbidities such as obesity and overweight may elevate the risk of adverse hepatic outcomes (Fan et al., 2024; Rinella et al., 2023; Targher, Tilg & Byrne, 2021). Our BMI-stratified analysis revealed significant intergroup differences (*P* < 0.05) in CAP, LSM, PT, ALT, AST, GGT, UA, GLU, HDL, HBsAg, HBeAg, and the non-invasive indices including APRI, GPR, and AAGP. Our data demonstrate that overweight and obese individuals bear greater hepatic metabolic stress, with elevated risks of lipid accumulation, inflammation, fibrosis, and concurrent metabolic dysregulation (*e.g.*, hyperglycemia, hyperuricemia). Previous studies confirmed that overweight and obesity not only increased the incidence of NAFLD but also worsened metabolic dysfunction and hepatic injury (Tang et al., 2023). A Korean cohort verified significant weight-dependent associations with elevated liver enzymes, metabolic abnormalities, and heightened fibrosis risk (Sohn et al., 2022). Wang et al. (2019) reported that each 1 kg/m^2^ BMI reduction increased ALT normalization likelihood by 38% (OR = 0.38, 95% CI [0.19–0.77]) in CHB patients. Rui et al. (2024) identified BMI as a key predictor of moderate-to-severe hepatic inflammation (AUROC = 0.86) in CHB patients with steatosis. These findings align well with our current results. This study found that the median quantitative HBsAg level was highest in Group 1 and lowest in Group 2. Weight gain may affect HBV activity through chronic inflammation or immune dysregulation. However, no differences in HBV DNA levels were observed among BMI groups; based on current research, there is no unified conclusion regarding the impact of BMI on serological virology (Kumada et al., 2021; Li et al., 2021; Yi et al., 2024).

Among enrolled patients, 80.8% (361/447) exhibited significant hepatic inflammation (G ≥ 2), with histological severity showing significant positive correlation with BMI stratification. Progressive BMI elevation corresponded to worsening inflammation, with the proportion of severe liver tissue inflammation (G4) reaching 8.9% in obese patients, significantly higher than the 1.2% in the normal weight group. These findings establish overweight/obesity as critical determinants of exacerbated inflammatory activity in MAFLD-HBV coinfection, suggesting their role as key progression risk factors. Clinical management should combine antiviral therapy with targeted weight interventions.

Multivariate regression analysis showed that LSM, BMI, HBV DNA, and ALT were independently associated with significant hepatic inflammation progression in patients with MAFLD and HBV infection. Hepatic fibrosis, a pathological alteration characterized by excessive extracellular matrix (ECM) deposition during chronic liver injury repair, exerts feedback effects on liver inflammation through three primary mechanisms: (1) ECM accumulation disrupts hepatic architecture, causing regional hypoxia and hepatocyte damage that perpetuates pro-inflammatory signaling; (2) Activated hepatic stellate cells and Kupffer cells secrete inflammatory mediators (TNF-α, IL-1β, *etc*.), creating a “fibrosis-inflammation” vicious cycle; (3) Certain ECM components possess intrinsic pro-inflammatory activity (Hammerich & Tacke, 2023). In summary, hepatic fibrosis influences inflammatory responses through multidimensional mechanisms involving cellular activation, mechanotransduction, oxidative stress, gut microbiota, and immune regulation, ultimately establishing a self-perpetuating “inflammation-fibrosis-inflammation” vicious cycle that exacerbates hepatic inflammation (Hammerich & Tacke, 2023). Obesity, as a primary NAFLD driver, independently promotes hepatic inflammation *via* adipotoxicity, inflammatory pathway activation, and gut dysbiosis-regardless of metabolic syndrome comorbidity, through core mechanisms involving adipokine dysregulation, oxidative stress, and immune homeostasis disruption (Wei et al., 2025). Obesity similarly exacerbates liver inflammation in CHB patients through multiple pathways encompassing lipotoxicity, immune dysregulation, and metabolic disturbances (Ellulu et al., 2017; Gu et al., 2025). The mechanistic interplay between obesity and MAFLD-HBV coinfection remains incompletely understood, warranting further investigation. HBV DNA reflects viral replication activity and antigen burden, and persistent replication can induce hepatocellular injury and promote necroinflammatory activity *via* immune-mediated mechanisms (Chen et al., 2022; Osmani et al., 2025; Zhao et al., 2024; Zoulim et al., 2024). Direct histological evidence in populations with concomitant MAFLD and HBV infection is currently limited. However, from a pathophysiological perspective, steatosis and metabolic dysregulation–induced chronic low-grade inflammation may alter the hepatic immune microenvironment, thereby amplifying the proinflammatory effects of HBV DNA (Osmani et al., 2025; Tourkochristou et al., 2022). In the present study, HBV DNA remained independently associated with hepatic inflammation in multivariate analysis, providing clinical support for the proposed mechanisms. In contrast, quantitative HBsAg levels and HBeAg status did not demonstrate independent predictive value for hepatic inflammation in this study.

ALT is a specific marker of hepatic inflammation and hepatocellular injury and an important biomarker for diagnosing liver inflammation in patients with CHB complicated by fatty liver disease, rather than a biological driver directly involved in the initiation or progression of inflammation (Burra et al., 2025; Fang et al., 2024). Therefore, ALT should not be interpreted as equivalent to upstream risk factors such as metabolic burden or the degree of liver fibrosis. The statistical significance of ALT observed in multivariate regression models should be interpreted as reflecting its marker-based association with, and predictive value for, the severity of hepatic inflammation rather than a causal contribution. Nevertheless, as one of the most commonly used and readily available biochemical parameters in clinical practice, ALT can still serve as a non-invasive surrogate indicator of hepatic inflammatory activity when histological data are unavailable, aiding preliminary risk assessment and clinical stratification in patients with MAFLD and HBV infection.

While hepatic steatosis induces mitochondrial dysfunction, ROS overproduction, and inflammatory cascade activation (Targher, Tilg & Byrne, 2021), our study found that CAP was not an independent risk factor for inflammation in MAFLD-HBV coinfection. This suggests liver inflammation progression in CHB-FLD may be predominantly driven by viral activity, metabolic dysregulation, and immune microenvironment alterations rather than mere steatosis (Hur et al., 2025). CAP, as a steatosis surrogate, may inadequately capture these complex interactions in our MAFLD-HBV cohort.

This study has several limitations. First, as a single-center retrospective study restricted to hospitalized patients from a hepatology department, patients with hypertension, diabetes, or hyperlipidemia were underrepresented, and selection bias cannot be excluded. Moreover, because the cohort predominantly comprised Southern Min Chinese individuals with region-specific BMI distributions, the generalizability of our findings to other populations may be limited, although this factor is unlikely to have substantially influenced the conclusions. Second, the cross-sectional observational design precludes causal inference.

## Conclusions

This clinical study was conducted based on histopathological findings and demonstrated that BMI, LSM, and HBV DNA are independent risk factors for the progression of hepatic inflammation in patients with MAFLD and HBV infection, whereas ALT served as an independent non-invasive predictor of this outcome. These findings provide a novel non-invasive approach for clinicians to assess the degree of hepatic inflammation when liver biopsy results are unavailable. Future prospective studies in populations with MAFLD and HBV infection are warranted to further validate these findings by incorporating a broader range of metabolic features and to continue exploring the potential application of non-invasive diagnostic models in assessing the risk of significant hepatic inflammation.

## Supplemental Information

10.7717/peerj.21186/supp-1 Supplemental Information 1 Codebook for Categorical Variables All categorical variables and their numeric-to-text label conversions used in the statistical analyses.

10.7717/peerj.21186/supp-2 Supplemental Information 2 Raw Data

## Additional Information and Declarations

### Data Availability

The following information was supplied regarding data availability: The raw data is available in the Supplementary File.
