# Supplementary material for: Retrospective analysis of predictors for significant hepatic inflammation in metabolic-associated fatty liver disease with chronic hepatitis B
Source: PeerJ. 2026 May 6;14:e21186. doi: 10.7717/peerj.21186 (PMC13156954; doi:10.7717/peerj.21186)
Supplement: Supplemental Information 1 — All categorical variables and their numeric-to-text label conversions used in the statistical analyses. [file peerj-14-21186-s001.docx]

**Codebook for Categorical Variables in the Study Dataset**

| Variable Name | Numeric Code | Category |
| --- | --- | --- |
| G(Grading of Hepatic Inflammation) | 1 | G1, mild activity, characterized by limited focal portal inflammatory infiltrates without significant bridging necrosis. |
|  | 2 | G2, moderate activity, with more extensive inflammatory infiltrates and focal spotty or piecemeal necrosis accompanied by mild interface hepatitis. |
|  | 3 | G3, severe activity, presenting with marked portal inflammation and frequent bridging or confluent necrosis. |
|  | 4 | G4, very severe activity, with widespread bridging necrosis or multilobular necrosis involving extensive areas of the liver. |
| HBeAg status (positive/negative) | 1 | Negative(＜1COI) |
|  | 0 | Positive(≥1COI) |
